# Comparative Longevity of Men and Women

**Published:** 1886-12

**Authors:** 


### COMPARATIVE LONGEVITY OF MEN AND WOMEN.

Interesting researches concerning the comparative longevity of men and women in Europe have recently been made by the Director of the Bureau of Statistics at Vienna. From these it appears that about a third more women than men reach advanced age. Women oftener lead quiet, regular lives. They have fewer bad habits, are less exposed to strong passion and excitement. It appears from the gathered statistics of the world that women have a greater tenacity of life than men. Nature worships the female in all its varieties. Among insects the male perishes at a relatively early period. In plants the seminate blossoms die earliest, and are produced in the weaker limbs. Female quadrupeds have more endurance than males. In the human race, despite the intellectual and physical strength of man, the woman endures longest, and will bear pain to which the strongest man succumbs. Zymotic diseases are more fatal to males, and more male children die than females. Deverga asserts that the proportion dying suddenly is about 100 women to 780 men ; 1,000 men in the United States committed suicide to 285 women. Intemperance, apoplexy, gout, hydrocephalous, affections of the heart or liver, scrofula and paralysis are far more fatal to males than females. Pulmonary consumption, on the other, hand, is more deadly to females, which argues we ought to give the girls of our families all the out-door exercise that they need. Females in cities are more prone to consumption than in the country. All old countries not disturbed by emigration have a majority of females in the population. In royal families the statistics show more daughters than sons. The Hebrew woman is exceptionally long-lived, while the colored man is exceptionally short-lived. Dr. Hough remarks that there are from two to six per cent. more males born than females, yet there is more than six per cent. excess of females in the living population. The rush and worry of the average business man in this country is apt to make him prematurely old, unless he takes judicious recreation, which he very seldom does. The females are, to a great extent, exempt from this overstraining about business cares, which may, in a degree, account for their superior vitality.—*The Indicator.*

